# Acceptance and feasibility of novel staple foods among individuals with type 2 diabetes in Singapore: a mixed methods study

**DOI:** 10.3389/fnut.2025.1594890

**Published:** 2025-06-11

**Authors:** Hafizah Yusri, Sean Jun Leong Ou, Dimeng Yang, Marcus Ting, Wei Lin Liew, Salome A. Rebello, Chin Meng Khoo, E. Shyong Tai, Mei Hui Liu

**Affiliations:** ^1^Department of Food Science & Technology, National University of Singapore, Singapore, Singapore; ^2^Saw Swee Hock School of Public Health, National University of Singapore, Singapore, Singapore; ^3^Department of Medicine, Yong Loo Lin School of Medicine, National University of Singapore, Singapore, Singapore

**Keywords:** staple foods, type 2 diabetes, continuous glucose monitoring, consumer perceptions, interviews, mixed method analysis

## Abstract

**Introduction:**

Novel staple foods (NVSFs) are defined as modified staple foods that provide healthier alternatives to type 2 diabetes (T2D) patients aiming to improve postprandial glycemic responses. We explored the expectations and perceptions of participants with T2D toward NVSFs alongside their feasibility using a mixed methods approach.

**Methods:**

Three distinct NVSFs were investigated: (i) bread fortified with anthocyanins for carbohydrase inhibition; (ii) white rice fortified with fiber; and (iii) microfluidic gel noodles reduced in available carbohydrates. Sixteen individuals with T2D participated in a 5-week crossover study where NVSFs or control staple foods were consumed in mixed meals for breakfast, lunch, and dinner. Participants wore continuous glucose monitoring devices during interventions to measure postprandial glycemic responses and interviews were conducted before and after interventions.

**Results:**

Qualitative analysis from interviews identified participants’ prior dietary choices, prior NVSF perceptions, expectations, and impressions, trade-offs between sensory and healthfulness, price, modification methods, and other socio-economic regular consumption considerations as key factors contributing to the acceptance of NVSFs. Among the NVSFs, the anthocyanin-fortified bread and fiber-fortified rice were preferred for their palatability, but not the microfluidic noodles. However, quantitative analysis from continuous glucose monitoring revealed only the microfluidic noodle meal demonstrating a significant reduction in 2-h iAUC value relative to its control from 160.2 ± 17.6 to 114.3 ± 12.2 mmol/L.min (mean ± SEM) *p* = 0.046.

**Discussion:**

This highlights the challenge of attaining a balance between health and favorable sensory properties for these NVSFs, which may be attributed to their modification methods. We conclude that an alternative staple food with palatable taste and texture properties modified through the reduction of available carbohydrates may be an effective approach to enhance the acceptance and feasibility of modified staple foods for T2D management.

## 1 Introduction

The number of adults who are living with diabetes worldwide is projected to increase to 643 million by 2030 (International Diabetes Federation, (1). In Singapore alone, the number is expected to increase to 872,300 and 898,900 in 2030 and 2045, respectively (International Diabetes Federation, (1). Diabetes is a major growing global burden, and dietary interventions remain a key factor in the management of diabetes (2, 3).

Staple foods, often sources of carbohydrates, have been associated with an increased risk of T2D. This association is partly due to the declining quality of carbohydrates in these foods over the years, driven by a growing shift in consumption toward high glycemic index (GI) refined carbohydrates (4–6). On the contrary, recent research has shown that the adoption of a low GI and low glycemic load (GL) diet is effective in slowing down diabetes progression (7).

In most Asian populations, carbohydrate-rich staple foods like bread, rice, and noodles are commonly consumed (8). According to the Singapore National Nutrition Survey 2022, wholegrain consumption among Singaporean adults remains significantly low with only 4% of staples consumed being wholegrain, in comparison to the recommended 30% despite governmental efforts (9). However, sensory properties of wholegrain staple foods have been reported to be less preferable (10) and described as bitter, coarse, and dry (11). Other factors playing a role in determining their consumption also include its higher cost, limited availability, and accessibility (12, 13). Moreover, refined carbohydrate staples have been integral to many local delicacies, and their substitution with wholegrains can alter the taste and overall culinary experience of these various meals. Thus, considerable barriers continue to hinder the consumption of these wholegrain staple foods.

The onset of complications arising from diabetes has also been associated with the postprandial glycemic responses of foods (14). These responses have been shown to be reliable predictors of long-term health implications (15). As carbohydrate-rich staple foods like bread, rice, and noodles have a direct influence on the postprandial glycemic response of individuals, many of these staple foods have been modified in an attempt to improve their postprandial glycemic responses, while striving to retain the sensory properties of conventional refined staples. These modified staple foods may be beneficial when incorporated into a low GI and GL diet for managing blood glucose levels. However, it is essential to thoroughly investigate their postprandial glycemic effects and make accurate claims to ensure they are appealing and beneficial for individuals with diabetes.

Novel staple foods (NVSFs) are designed with the objective of improving post-prandial glycemic responses without significantly altering dietary preferences. While individuals with T2D have been reported to be more willing to compromise on the taste and cost of food (16), data on lived experiences involving the incorporation of these NVSFs in the diet remain largely unclear among them in the context of a Southeast-Asian population.

In the present study, three types of NVSFs were employed and consumed as components of mixed meals for breakfast, lunch, and dinner respectively: (i) anthocyanin-fortified bread (17); (ii) fiber-fortified rice; and (iii) microfluidic gel noodles (18). Bread was modified by fortification with anthocyanins for the inhibition of carbohydrases, rice was modified by fortification with fiber to reduce the rate of digestion, and noodles was modified through the reduction of available carbohydrates.

As such, this study aims to (1) explore the expectations and perceptions of individuals with T2D toward NVSFs, and (2) examine their feasibility to serve as healthier staple food alternatives from their experiences and the effect on their postprandial glycemic responses.

## 2 Materials and methods

### 2.1 Study design

**Figure 1:**
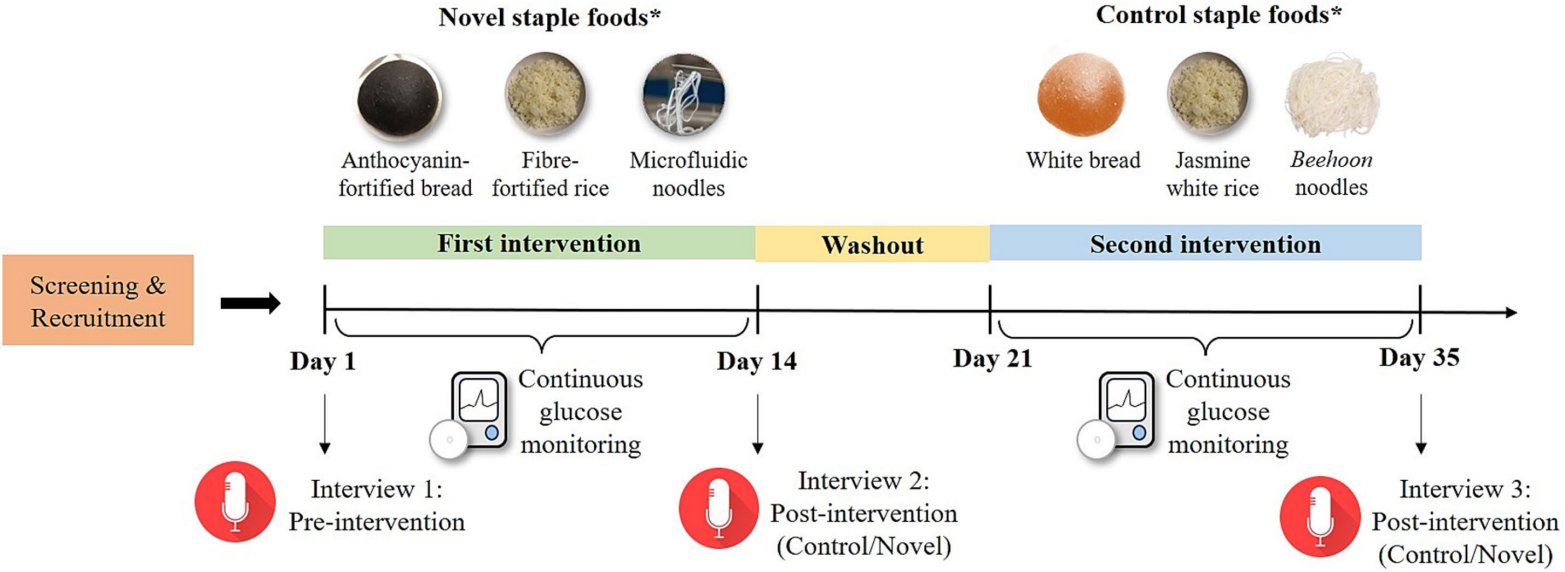
Overall study schematics and physical appearance of each NVSF and their control counterparts. *Participants were randomly assigned to novel or control intervention for the first treatment, followed by the other intervention after 1 week of washout.

This study was a 5-week randomized controlled, within-subject crossover study, consisting of two 2-week intervention periods with a 1-week of washout in between (Figure 1). For each intervention period, participants were instructed to consume NVSFs (novel) or control staple foods (control) in mixed meals for breakfast, lunch, and dinner. The sequence of intervention for each participant was generated using a randomization software. For each participant, a total of three one-to-one interview sessions were conducted before the study commencement and after each intervention period. Continuous glucose monitoring was also employed during both intervention periods. Additionally, food diaries were provided for each intervention where participants were instructed to annotate the time at which each test meal was consumed during the day, and for any snacks which were consumed in between meals. Participants were also advised to consume the test meals within 15 min and to fast 2 to 3 h prior to consuming the test meals and after consumption to ensure accuracy of their postprandial glycemic responses. They were allowed to snack on foods of their choice after the fasting window, with the exception of anthocyanin-containing foods or supplements, which they were required to abstain from for at least 1 week before the study period. They were instructed to take a photograph of their empty food container after each meal and send it to a study coordinator to verify their compliance with the meal plan. This study was approved by the National Healthcare Group Domain Specific Review Board (Reference No. 2019/00952) and registered at ClinicalTrials.gov (NCT05399134). Participants gave written informed consent before enrolling into the study.

### 2.2 Participants

**Figure 2:**
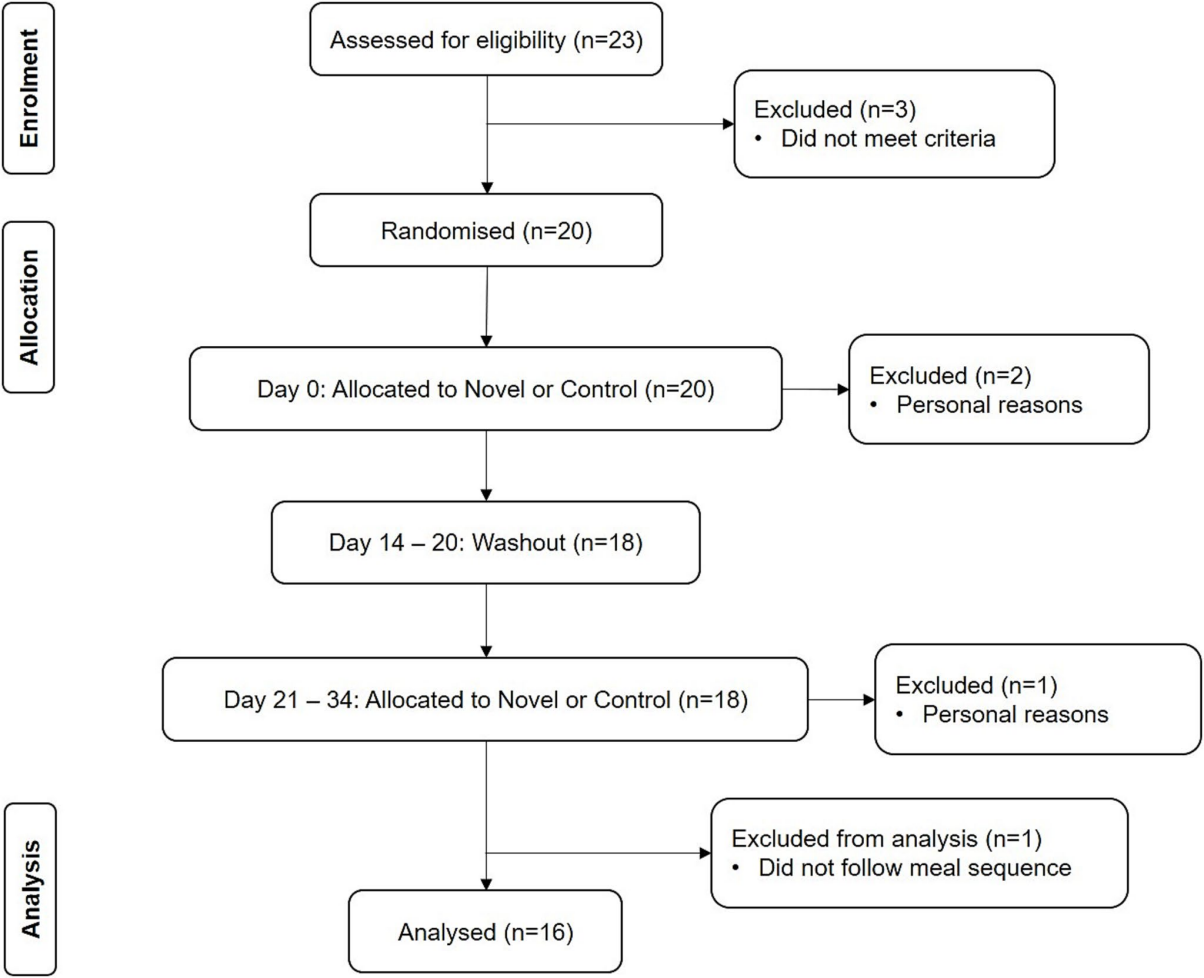
CONSORT diagram.

Twenty Chinese participants with T2D were enrolled into the study. Recruitment was conducted via poster advertisements displayed at the National University Hospital, Singapore, and through local newspaper. Participants initially approached study coordinators to express their interest through a phone call and/or email. Participants who met the recruitment criteria were eligible to attend a screening session conducted at the Department of Medicine, National University of Singapore. The recruitment criteria excluded participants who were smokers, those receiving prandial insulin therapy, individuals with unstable medical conditions, and those who had allergies to the test meals. Inclusion criteria included participants who were English-speaking, aged between 21 and 70 years old, and clinically diagnosed with T2D with a HbA1c of less than 10%. Three of the participants dropped out due to personal reasons, and one participant was excluded from analysis as the participant did not follow the meal sequences provided. The remaining 16 participants completed the study and were included for analysis. Figure 2 portrays the Consolidated Standards of Reporting Trials (CONSORT) diagram of the participants in the study.

### 2.3 Test meals

Each staple food was served with standardized side dishes in mixed meals for breakfast, lunch, and dinner (Table 1). Microfluidic gel noodles were consumed for dinner every other day and were replaced with control *beehoon* (rice vermicelli) noodles for the other days throughout the 2-week novel intervention period. This was done with the aim of enhancing compliance with the microfluidic gel noodles, which were expected to be less palatable compared to the other two NVSFs. Chicken broth was also added to the microfluidic gel noodles to improve the overall meal’s palatability.

**Table 1:** Test meals provided for novel and control interventions.

Meal	Staple food		Standardized side dishes
	Novel	Control	
Breakfast	Anthocyanin-fortified bread	White bread	Soy milk reduced sugar (NutriSoy), extra virgin olive oil butter (SCS)
Lunch	Fiber-fortified rice	Jasmine white rice	Sesame & ginger stew chicken, sauteed spinach Or Oven baked chicken thigh with herbs, sauteed spinach
Dinner	Microfluidic gel noodles with chicken broth (Swansons)	*Beehoon* noodles	Sesame & ginger stew chicken, sauteed spinach Or Oven baked chicken thigh with herbs, sauteed spinach
	Or *Beehoon* noodles		

The jasmine white rice, fiber-fortified rice, *beehoon* noodles, and chicken and spinach side dishes were prepared by a food caterer licensed by the Singapore Food Agency (Creative Eateries Pte. Ltd.). Fiber-fortified rice was prepared by incorporating a novel fiber composition blend (5ibrePlus™, Alchemy Foodtech Pte. Ltd., Singapore) into uncooked white rice before cooking it with water. The quantity of fiber blend added was 20% of the mass of the uncooked rice. Soymilk reduced sugar (NutriSoy, Fraser and Neave Pte. Ltd., Singapore), extra virgin olive oil butter (SCS Dairy, DKSH Marketing Services Pte. Ltd., Singapore), and chicken broth (Swansons, Campbell Soup Company, New Jersey, United States) were purchased from a local supermarket (NTUC Fairprice Cooperative, Ltd., Singapore).

The anthocyanin-fortified bread, white bread, and microfluidic gel noodles were produced in a food processing facility at the Department of Food Science and Technology, National University of Singapore. Anthocyanin-fortified bread and white bread were prepared according to methods described by Sui, Zhang and Zhou (19) and Ou, Yu (20) (Supplementary material 1). Microfluidic gel noodles were produced following the techniques described by Lin, Yang (18) (Supplementary material 2). Microfluidic gel noodles are a novel food format produced via an extrusion process, where a soy protein isolate solution is encapsulated within a gel matrix formed by the interaction of sodium alginate and calcium chloride, resulting in noodle-like structures. Unlike commercial noodles, these gel-based noodles are engineered to promote satiety while eliciting a lower postprandial glycemic response.

Table 2 shows the nutritional compositions of each meal containing the staple foods. Nutritional compositions of the test meals were analyzed by an external laboratory (TÜV SÜD PSB Pte. Ltd., Singapore).

**Table 2:** Estimated nutritional composition of test meals.

		Breakfast		Lunch		Dinner	
Sex	Nutrients	Novel	Control	Novel	Control	Novel	Control
Male	Total energy (kcal)	593.85	593.85	482.98	469.86	251.77	451.21
	Protein (g)	17.70	18.15	42.26	41.94	41.67	40.11
	Fat (g)	17.45	17.45	5.46	5.78	6.45	13.41
	Carbohydrate (g)	97.65	95.40	79.56	72.52	15.80	50.84
	Available carbohydrates (g)	91.50	91.05	66.20	62.52	6.76	42.52
	Fiber (g)	6.15	4.35	13.36	10.00	9.04	8.32
Female	Total energy (kcal)	593.85	593.85	350.01	340.17	182.40	381.84
	Protein (g)	17.70	18.15	29.76	29.52	30.57	29.01
	Fat (g)	17.45	17.45	3.81	4.05	4.56	11.52
	Carbohydrate (g)	97.65	95.40	59.04	53.76	12.48	47.52
	Available carbohydrates (g)	91.50	91.05	49.17	46.41	4.77	40.53
	Fiber (g)	6.15	4.35	9.87	7.35	7.71	6.99

Total energy of test meals for males were higher than females due to higher daily energy requirements. Total energy of the dinner novel meal was lower than the control meal due to reduction of available carbohydrates in microfluidic noodles.

### 2.4 Interviews

The interview sessions were conducted in a private room with an interviewer (the first author) and a note-taker (the third author). All authors had no prior relationships with the study participants. Interviews were conducted in English, audio recorded, and subsequently transcribed verbatim. Interview transcripts were qualitatively analyzed through an inductive approach. Initially, line-by-line coding was used to generate codes by closely examining the content of the transcripts. Codes that were related were then organized into sub-themes, and these sub-themes were further grouped into overarching themes by the first author. The themes were refined through consistent review and validation by the first, third, and sixth author before being finalized. Any discrepancies were discussed until a consensus was reached. NVivo (QSR International Pty Ltd., Melbourne, Victoria, Australia) was used for data management and organization.

A total of three interviews were conducted for each participant: (1) pre-intervention; (2) post-intervention (novel); and (3) post-intervention (control). The pre-intervention interview focused on the introduction to the NVSFs with the presentation of food samples. In contrast, the post-intervention interviews centered around gathering feedback on the staple foods, including participants’ thoughts on regular consumption considerations, cost considerations, any challenges faced, and their personal experiences with diabetes. The interview guide was co-developed by the third and sixth author with a key focus on understanding participant’s lived experiences with the foods in reference to Lim, Ting (16). A sample of the interview guide is shown in Supplementary material 3, Table S1. The qualitative data is reported following the Consolidated Criteria for Reporting Qualitative Studies (COREQ) guidelines.

### 2.5 Continuous glucose monitoring

Continuous glucose monitoring (CGM) was conducted during both 2-week intervention periods. Before the start of each intervention, a CGM device (FreeStyle Libre Pro, Abbott Diabetes Care Inc., Netherlands) was inserted at the back of the participant’s upper arm following instructions provided by the manufacturers. The CGM device was removed after the intervention period and scanned using a reader to retrieve the raw glucose readings recorded over the 2-week period. Subsequently, the 2-h postprandial glucose readings for the meals were extracted, based on the times of meal consumption reported by the participants in their food diaries. The incremental area under the curve (iAUC) over the 2-h postprandial period was then calculated. The iAUC quantifies the total rise in glucose concentration above the pre-meal (fasting) baseline, providing a measure of the net postprandial glycemic excursion attributable to each test meal.

### 2.6 Sample size

Sample size calculation was based on a study conducted by Rieder, Knutsen (21), which reported a significant decrease in iAUC for white bread when fortified with 3.8 g of beta-glucan fiber from 165.8 ± 20.4 to 106.8 ± 13.9 mmol/L.min (mean ± SEM). Using a 0.05 significance level (*α* = 0.05) and a power of 0.8, at least 16 participants were required to observe a reduction in iAUC after staple food fortification.

The sample size for the qualitative aspect of the study was guided by the principle of saturation which describes a point at which further data collection would not yield additional insight (22). Based on studies by Hennink, Kaiser and Marconi (23) and Hennink and Kaiser (24), ‘meaning saturation’ which relates to a comprehensive understanding of issues raised in interviews, required 16 to 24 interviews. Based on this range and the sample size calculation above, a minimum of 16 participants were required for this study. To account for attrition, a total of 20 participants were recruited.

### 2.7 Statistical analysis

Postprandial glycemic responses were presented as 2-h iAUC. Data was checked for normality using the Shapiro–Wilk test before performing a paired *t*-test to analyze for any differences in 2-h iAUC values between the novel and control meals. Significance was defined as *p*-value < 0.05. Additionally, 95% confidence intervals (CI) were calculated for the mean differences between meals. Effect size was measured using Cohen’s *d*, calculated as the mean difference between the two groups divided by the pooled standard deviation. Cohen’s d values were interpreted as small (0.2), medium (0.5), or large (0.8). The difference in postprandial blood glucose concentrations from baseline over 2 h was also presented as a ‘change in glucose’ graph. GraphPad Prism Version 8.0.2 (GraphPad Software Inc., California, United States) was used to conduct the statistical tests. Values were presented as mean ± standard error of mean (SEM) unless otherwise stated.

### 2.8 Mixed methods analysis

A convergent parallel structure was utilized for this mixed methods study, where the qualitative and quantitative data were separately analyzed before they were merged for interpretation to allow for a cross-validated understanding of the study results for each of the NVSFs (25, 26). Both datasets were integrated together following a four-step approach by Skamagki, King (27) which encompasses (1) the creation of a joint display table; (2) linking activity; (3) establishing relationships; and (4) interpretation of data. Qualitative and quantitative datasets for each NVSF were merged using this approach to derive meaningful interpretations regarding the acceptance and feasibility of these NVSFs.

## 3 Results

### 3.1 Participants

Twenty participants diagnosed with T2D were enrolled into the study. Of these participants, three dropped out due to personal reasons. One participant was excluded from analysis due to non-compliance while the remaining 16 participants completed the study and were included for analysis. These participants were also asked about the types of staple foods commonly consumed in their households. Majority of the participants reported more than one type of staple food typically being consumed in each household (i.e., bread, rice, and noodles). The baseline characteristics of the participants are shown in Table 3. The weight and HbA1c of participants across the study timepoints were also measured and presented in Supplementary material 4, Table S2, however there were no significant changes.

**Table 3:** Baseline characteristics of participants (*n* = 16).

Characteristics	Mean ± SD or N (%)
Age (years)	57.9 ± 9.45
Gender	
Male	12 (75%)
Female	4 (25%)
Weight (kg)	71.5 ± 21.4
BMI (kg/m^2^)	26.1 ± 6.26
HbA1c (%)	7.23 ± 0.65
Commonly consumed staple food in household	
Bread	7 (43.75%)
Rice	13 (81.25%)
Noodles	11 (68.75%)
No. of years living with diabetes	
1 to 5 years	6 (37.5%)
6 to 10 years	3 (18.75%)
More than 10 years	7 (43.75%)

### 3.2 Qualitative findings

From the qualitative analysis of interviews, seven key themes were identified related to the acceptance of NVSFs: prior dietary choices as individuals with T2D; prior perceptions and expectations of NVSFs pre-tasting; visual first impressions of NVSFs; trade-off between sensory and health properties; price and willingness to pay; modification methods; and other socio-economic factors for regular consumption. A summary of themes, sub-themes, and their illustrative quotes are presented in Table 4 for participants’ initial perceptions of NVSFs and their dietary choices prior to the study, and Table 5 for their perceptions post-intervention.

**Table 4:** Summary of themes, sub-themes, and illustrative quotes of qualitative findings of participants’ dietary choices prior to the study and their initial perceptions, expectations, and impressions of NVSFs.

Theme	Sub-themes	Illustrative quotes
Dietary choices as individuals with type 2 diabetes prior to study	Strategies to control dietary intake	“I tell myself I must control my diet. I do not like to eat sweet things and I do not like to eat a lot of portions of rice, in fact only brown rice. Just half a bowl or below half.” – M, female, 64 years old
	Difficulty in maintaining healthy diet	“Keeping an eye on the kind of food that I take is also a hassle. I just cannot eat as and when I like. that is just an inconvenience actually.” – MT, male, 58 years old
	Difficulty in trading sensory properties for health	“There is no such thing that is sweet and then the glucose level would not increase, so everything got a price to pay for it, right?” – M, male, 69 years old
Prior perceptions and expectations of NVSFs pre-tasting	Conceptual understanding as modified foods for health purposes	“Sounds like it’s being modified, or they add probably things inside the ingredients. Yeah, they modify the ingredients, they add something extra. Probably they are trying to find out you know whether that will help to bring down the glycemic level, so they need to modify it.” – ML, female, 63 years old
	Sensory considerations	“My expectation, my own version, may be not as tasty as your grandmother’s or your mother’s food you know, because they got put a lot of MSG (monosodium glutamate), you know salt, and sugar, and everything. Whereas basically [NVSFs] will be quite bland.” – L, male, 48 years old “I think [the taste] should be the same as normal staple food.” – MT male, 58 years old “Maybe not for everything, maybe some [NVSFs] they are able to do [the appearance] almost the same but some they are still trying to make it similar.” – MG, male, 52 years old “I’m not particular about appearance.” – Mr. Soo, male, 62 years old
	Higher price anticipated due to special production methods	“I would think that [the price] is slightly higher. I would think slightly higher since some work has been done on this food.” – A, male, 55 years old
	Little experiences with NVSFs	“Well, most of the bread these days they add vitamins right. You can see that. Fortified, those are fortified. Maybe not [consumed] a lot, but a couple of times a month maybe.” – S, male, 43 years old
	Prior experiences with NVSFs	“I’m quite ok with [NVSF]. In fact, I have been taking such food, I mean more or less. It’s a healthy food. So long it is healthy then I would just consume. I mean they can make it nicer in whatever way, but I do not think it tastes that bad. Probably it’s a bit more course, that’s probably all. It depends on how you cook it. It’s positive [experience].” – S, male, 55 years old
	Staple food most preferred to be modified: Bread (4) Rice (11) Noodles (7)	“I think usually rice, noodle, *beehoon* all these. All these are the very common foods that I eat every day, for everyone.” – M, female, 64 years old
Visual first impressions of NVSFs	NVSFs associated and compared with commonly consumed foods	Bread “[Anthocyanin-fortified bread] make me feel like it’s made from *pulut hitam* (black glutinous rice).” – M, female, 64 years old Rice “This one is normal. Anyway, I do not take. I do not take white rice.” – MT, female, 66 years old Noodles “No different from the regular *beehoon* that I’m consuming. Not much difference to me.” – A, male, 55 years old
	Initial impression of unconventional colors	“[Anthocyanin-fortified] bread looks a bit weird. I mean I have not seen bread that is completely black. Yeah, I think I will not take it if it’s like that. Yeah, the color.” – MT, male, 58 years old
	Initial impression of unusual textures	“Appearance [of microfluidic noodles] looks a bit odd-looking. I believe I’m not the only one who said that right?” – S, male, 55 years old “I do not know what you call this noodle. Looks like some white colored rubber bands?” – S, male, 43 years old

**Table 5:** Summary of themes, sub-themes, and illustrative quotes of qualitative findings from post-intervention interviews.

Theme	Sub-themes	Illustrative quotes
Trade-off between sensory and health properties	Blood glucose control motivational factor for consumption	“The motivation will be to help me to control my blood sugar. With this type of food, I think we can live much longer and healthier.” – MG, male, 52 years old “I hope that these modified foods can reduce my sugar level so that I can do away with the medicine that I’m taking.” – A, male, 55 years old
	Perceived improvement in blood glucose levels	“Every time after eating I do my own [glucose] measurement. … The spike is not so high. … Normal foods before this… it spikes very high. [NVSFs] at least sort of keep it in control.” – MH, male, 65 years old
	Color of NVSF associated with health properties	“I am sure that the [fibre-fortified] rice and probably the brown rice is similar in health quality. For the [anthocyanin-fortified] bread you cannot find it.” – MG, male, 52 years old “To me I feel that it’s quite natural in a sense because it’s like made of plant extract, it’s considered healthy.” – MN, female, 35 years old
	Backed by scientific evidence	“I do not have any concerns if I know that given to me that these are good for you. The benefits, and it’s been scientifically based. Then no issue.” – MT, female, 66 years old
	Taste and texture crucial for continued usage	“I do not like [the microfluidic gel noodles], very artificial, when you chew on it, it’s like chewing on plastic.” – L, male, 48 years old “[Fibre-fortified rice] there’s not much difference to the white rice, so it’s very easy to actually accept it.” – MN, female, 35 years old
	Role in diabetes management	“[NVSFs] gives more choice to diabetic patients and also other people who are health conscious. So yeah, I think this is a very good change for everyone.” – MN, female, 35 years old
	Partial adoption	“I probably will not [consume] on a very consistent basis, but as a novelty. As a novelty, you eat it once in a while. It’s not like something that you reach out for.” – J, female, 57 years old
Price and willingness to pay	Expected to cost more	“There’s some certain value-added process in it, and definitely the cost of making all this will be more than the usual staple food. So therefore, I think it’s only fair. I think they need to increase a bit. But in the case of driving this message across, or to increase the sales, they should not start with the higher cost. But eventually the cost has to go up. Because if not, how can we survive all the R&D inside there?” – H, male, 67 years old
	Willingness to pay more for health benefits	“I mean, it really provides health improvement. I do not mind paying a slight premium for that. … I mean if everything else being it’s safe and it’s got a low GI value.” – MT, male, 58 years old “Preferably from consumer’s point of view it really should not be above what is normal to make it affordable, easily available.” – MT, female, 66 years old
	Consideration to lower price for special groups	“Being a senior, the price should be like the basic normal staple food price, just for seniors. Because you know we have got no more income, and it should supplement us.” – MH, male, 65 years old
Modification methods	Smaller sensory changes for NVSFs modified via fortification preferred	“[Anthocyanin-fortified bread] tastes generally like bread anyway, not much difference from any other bread that I’ve tasted.” – H, male, 67 years old “[Fibre-fortified] rice there’s not much difference to the white rice, so it’s very easy to actually accept it.” – MN, female, 35 years old
	Greater sensory changes for NVSF modified via carbohydrate reduction	“I find that [the microfluidic gel noodles] is too hard and even despite the time that I cook it, I put it to boil to ten fifteen, even up to thirty, it’s still very tough. I just cannot bring myself to buy or eat this noodle, even at what cost.” – A, male, 55 years old
Other socio-economic factors for regular consumption	Considerations from family members	“As a family, we eat together the same food for that meal. If not, we’ll have to do two cookings. If I stay alone with my wife maybe it’s ok, two people. The management of the family having a meal together. I think that’s a bit difficult to arrange.” – F, male, 63 years old
	Recommendations need to be provided on ways to consume	“Of course, there must be some suggestions of ways to eat. Maybe they can say, ‘It will be tasty if you put this butter, or you know something’, so that at least people know how to consume it.” – MG, male, 52 years old
	Increase in accessibility	“Now HDB (i.e., apartment) blocks you can have a 24-h vending machine. That will be good. It has to be within easy reach. I mean, you cannot expect an old person to travel for half an hour. They could be at a block you know, a void deck.” – MS, male, 62 years old

#### 3.2.1 Dietary choices of individuals with T2D prior to study

While participants recognized the benefits of physical activity in diabetes management, dietary interventions were greatly emphasized. Many participants described becoming more aware of the metabolic effects of foods after their diabetes diagnosis. This motivated many of them to restrict their carbohydrate and sugar intake as a strategy to control their blood glucose levels.

“Of course, I do not mind taking carbohydrates, but I abstain because I know it’s going to cause my glucose level to go up. Therefore, I do not take carbohydrate at all… We got to abstain from carbohydrates. It’s the most important step first.” – M, male, 69 years old.

However, participants also shared challenges they commonly encountered while trying to adhere to healthy dietary behaviors. These challenges stemmed from the necessity of maintaining consistency, the constraints on indulging in high-glycemic index foods freely, and the perception that ‘healthy’ foods often lacked in taste. Notably, participants found it difficult to compromise on sensory enjoyment for the sake of health considerations.

“To me diabetes is very troublesome and easily forgettable. Because I have a sweet tooth, so I love red beans, I love buns. All these refined sugars. so sometimes I just close one eye. Yeah, very difficult to manage.” – J, female, 57 years old.

Developing complications related to diabetes were also a source of worry for many participants and many expressed a desire to be less dependent on medication. These concerns were also impetus for their dietary changes, though the challenges encountered have made it difficult for them to be maintained.

“[I’m] trying not to depend on medicine. I know I inject insulin can bring back [glucose] to normal but I cannot be doing insulin for the rest of my life, I do not want that.” – O, male, 62 years old.

#### 3.2.2 Prior perceptions and expectations of NVSFs pre-tasting

Many participants viewed NVSFs conceptually as staple foods adapted for health purposes or to enhance the nutritional capacity of the staple foods. Particularly, NVSFs were regarded as being fortified with ‘good’ ingredients that would aid in glycemic control for diabetes management. However, there were several participants who regarded NVSFs as genetically modified and processed foods, and as a new food innovation that has not been tested or commercially available in the market.

“Novel, to my understanding is something new, something that nobody has come up with. Staple food is rice and all those things. Novel staple foods mean it’s a new thing that maybe the scientist is trying to discover.” – MG, male, 52 years old.

Majority of the participants expected NVSFs to taste and appear similar to conventional staples, though their perceived health benefits also led some to believe that their taste will be compromised. Some participants also stated that the appearance of a food item is not a substantial factor in determining their inclination toward the food as compared to taste which holds a greater significance.

For pricing, participants generally expected the NVSFs to be priced higher than the control staple foods. The rationale behind these higher prices originated from anticipated special production costs associated with NVSFs and the use of premium ingredients to produce them.

“Better ingredients come with a higher price. I think that is fair. There is no way that you can sell at the same price, no way.” – MS, male, 62 years old.

Participants also reported little experiences with NVSFs, citing vitamin-fortified breads which were commonly consumed. Some also mentioned prior experiences with these fortified foods where they were usually consumed with other side dishes, enhancing their taste profile. However, modification of other staples (e.g., rice and noodles) were rarely mentioned. Additionally, when asked on their most preferred staple food to be modified, rice was named as the most popular staple food (11/16 participants), followed by noodles (7/16 participants) and bread (4/16 participants).

#### 3.2.3 Visual first impressions of NVSFs

During the pre-intervention interview, participants drew associations between the NVSFs and commonly consumed foods, using them as a point of reference. The anthocyanin-fortified bread was often compared to familiar local foods like *pulut hitam* (black glutinous rice) and charcoal bread (black bread containing activated charcoal), while the microfluidic gel noodles was associated with local Asian *beehoon* and *mee sua* (wheat vermicelli) noodles, though they were also described as ‘odd-looking’. In contrast, majority of the participants observed no differences between the fiber-fortified rice and conventional white rice. However, their initial impression of the anthocyanin-fortified bread also garnered the most attention due to its black or dark purplish appearance. This presented a stark contrast to the typical appearance of conventional white bread which led to poor appeal from several participants.

“The [anthocyanin-fortified bread] is a bit surprising because it’s totally black. It looks a bit stale. Kind of not very appetizing.” – H, male, 67 years old.

On the other hand, the close resemblance between the fiber-fortified rice and conventional white rice had an unfavorable impact on some participants’ perceptions of the fiber-fortified rice. Their rationale was that ‘white’ rice may not be suitable for individuals with diabetes.

“No concerns, except that this [fibre-fortified rice] will not work for me. White rice is no, that’s personal for me.” – MT, female, 66 years old.

#### 3.2.4 Trade-off between sensory and health properties

Participants named health benefits as the most attractive feature of NVSFs in managing postprandial blood glucose responses. This motivated participants to consume NVSFs during the study and was a key consideration for future consumption.

“The only thing that will help me consume [the NVSFs] is if the glucose response is good. Other than that, I do not think I will eat this on a daily basis. If the glucose reading is improved, yes, I will make the switch.” – O, male, 62 years old.

Several participants also reported an improvement in postprandial glycemic responses based on their personal blood glucose measurement. This reaffirmed their confidence toward the perceived health benefits of NVSFs.

“I think for a diabetic patient, it is good news because I do [glucose] monitoring every morning. I can see that the blood glucose will not rise very high, so it’s within my expected range.” – MG, male, 52 years old.

Interestingly, the unconventional color of the anthocyanin-fortified bread was also later perceived as unique and healthy. This also enhanced their liking toward the bread and their perception of its healthfulness. Though, some participants also hoped there will be scientific evidence to substantiate the health benefits of NVSFs.

“I think it would be good if they can show proof that [the NVSFs] do help.” – ML, female, 63 years old.

However, taste and texture were emphasized as the primary sensory considerations for continued usage in post-intervention interviews. Generally, participants reported no notable differences in taste between the anthocyanin-fortified bread and fiber-fortified rice and their control counterparts, though there were various preferences when it came to the texture of bread and rice. Conversely, the microfluidic gel noodles received a significantly less favorable assessment from many participants. They described these noodles as ‘rubbery’, ‘plastic-like’, and ‘difficult to chew’, expressing a level of discontent with their taste and texture.

“The [microfluidic] noodles are tasteless basically. It’s like consuming plastic strings.” – L, male, 48 years old.

Additionally, as the standardized sides provided were repetitive throughout the intervention periods, a participant also emphasized the importance of having a variety of side dishes to complement NVSFs as taste was a significant factor affecting their perceptions of NVSFs (S, male, 43 years old).

Nevertheless, participants generally perceived NVSFs as playing a role in diabetes management. Participants felt that NVSFs offered diabetic-friendly staple food alternatives to conventional staples, allowing them to consume local favorites while simultaneously managing blood glucose levels.

“You can have a normal diet on a daily basis, no constraints… without carbo but with the benefit. The glucose is being managed.” – MT, female, 66 years old.

Despite this, most were only open to the partial adoption of NVSFs; incorporating NVSFs into their diet two to three times a week or every other day rather than daily.

“I think I’m ok [to consume] but not every day, so probably two or three times a week, it depends.” – S, male, 55 years old.

The type of NVSFs was also important. Many participants were not keen on incorporating the microfluidic gel noodles as part of their regular diet due to their discontentment with its taste and texture.

“I think it’s hard for the consumers to just buy the [microfluidic] noodles, unless there’s something so special about it that they can overlook.” – MT, female, 66 years old.

#### 3.2.5 Price and willingness to pay

After the study interventions, participants still expected the NVSFs to be priced higher than the control staple foods due to perceptions around their special production costs and use of premium ingredients.

“The price will be higher because the production will be more. The cost to produce this thing will be higher. That’s why it is more. Compared to a simple white bread [which] probably the cost is less. And also I think to get the certification for the [value] of GI all these… The way they produce it’s more complicated and so you have to pay more for the processing.” – MG, male, 52 years old.

However, the willingness to pay a higher price for these NVSFs exhibited significant variation among participants. Among those who expressed a willingness to pay more, many specified only a slight increase of 10–20% higher than the cost of the control staple foods, while others were willing to pay double the cost. Their willingness to pay more was contingent on the condition that the NVSFs offered health properties that were beneficial to them.

“Depends on the benefits I can get. If you do it even double the price, that’s the price that we pay as long as it can still benefit.” – M, male, 69 years old.

Conversely, among those who were not willing to pay a higher price for NVSFs, participants believed that the prices of NVSFs should be on par with the cost of the control staple foods to ensure affordability for most of the consumers. Other reasons cited for unwillingness to pay more included the need for lower prices particularly for seniors and individuals with low incomes who have diabetes, the availability of cheaper alternatives in the market that have already achieved similar health effects, and the belief that consuming smaller amounts of the better-tasting and more affordable conventional staple foods represented a more cost-effective solution compared to spending more on costlier NVSFs.

#### 3.2.6 Modification methods

Notably, participants reported smaller sensory changes for NVSFs modified by way of fortification (i.e., anthocyanin-fortified bread and fiber-fortified rice). These sensory changes were made in comparison to their control staple food counterparts. However, greater sensory changes were reported for the microfluidic gel noodles, which was modified via carbohydrate reduction.

“Actually, the [microfluidic] noodles is really bad. Very lumpy. I eat a lot of noodles but this is the worst noodles I’ve eaten. I will not consume it.” – MT, male, 58 years old.

Furthermore, when asked to select their preferred NVSF out of the three options, only the fiber-fortified rice and anthocyanin-fortified bread were chosen. The primary reasons for selecting these two NVSFs were their minimal differences in taste and texture when compared to their conventional staples, a characteristic which was lacking in the microfluidic gel noodles.

“I would purchase the [fibre-fortified] rice. I will not purchase the [microfluidic] noodles. The [anthocyanin-fortified] bread occasionally.” – A, male, 55 years old.

#### 3.2.7 Other socio-economic factors for regular consumption

Attitudes of family members toward the NVSFs also influenced participants’ continued consumption of the foods. Often, the support provided by family members motivated participants to maintain healthy dietary habits.

“My husband tells me every time, never give up, so because of him I have help to carry my food back. I tell myself. with my family’s support, my daughter’s support… I was trying my best to complete the whole study.” – M, female, 64 years old.

On the other hand, some participants viewed NVSFs as being specifically designed for people with diabetes, and did not wish for the rest of their household to be subjected to similar constraints and consume ‘special’ foods. Related to this, the household preparation of NVSFs just for themselves was viewed as being troublesome.

“My family are not diabetic. I do not think they need to undergo such a treatment.” – MH, male, 65 years old.

“Preparing all these at home will be quite clumsy for only one portion, one serving. Too little to prepare, too small to prepare.” – H, male, 67 years old.

In addition, participants also highlighted the need for recommendations on ways to consume the NVSFs and guidance on their preparation methods to ensure the seamless integration of NVSFs into their dietary routines. Accessibility of the NVSFs was also discussed, with lack of availability perceived as a barrier to regular consumption.

“Everywhere sells bread. You know, you walk less than 100 meters, you got a bread shop here, a bread shop there. Am I going to go to a special supermarket, just to buy your special bread?” – S, male, 43 years old.

### 3.3 Postprandial glycemic responses

**Figure 3:**
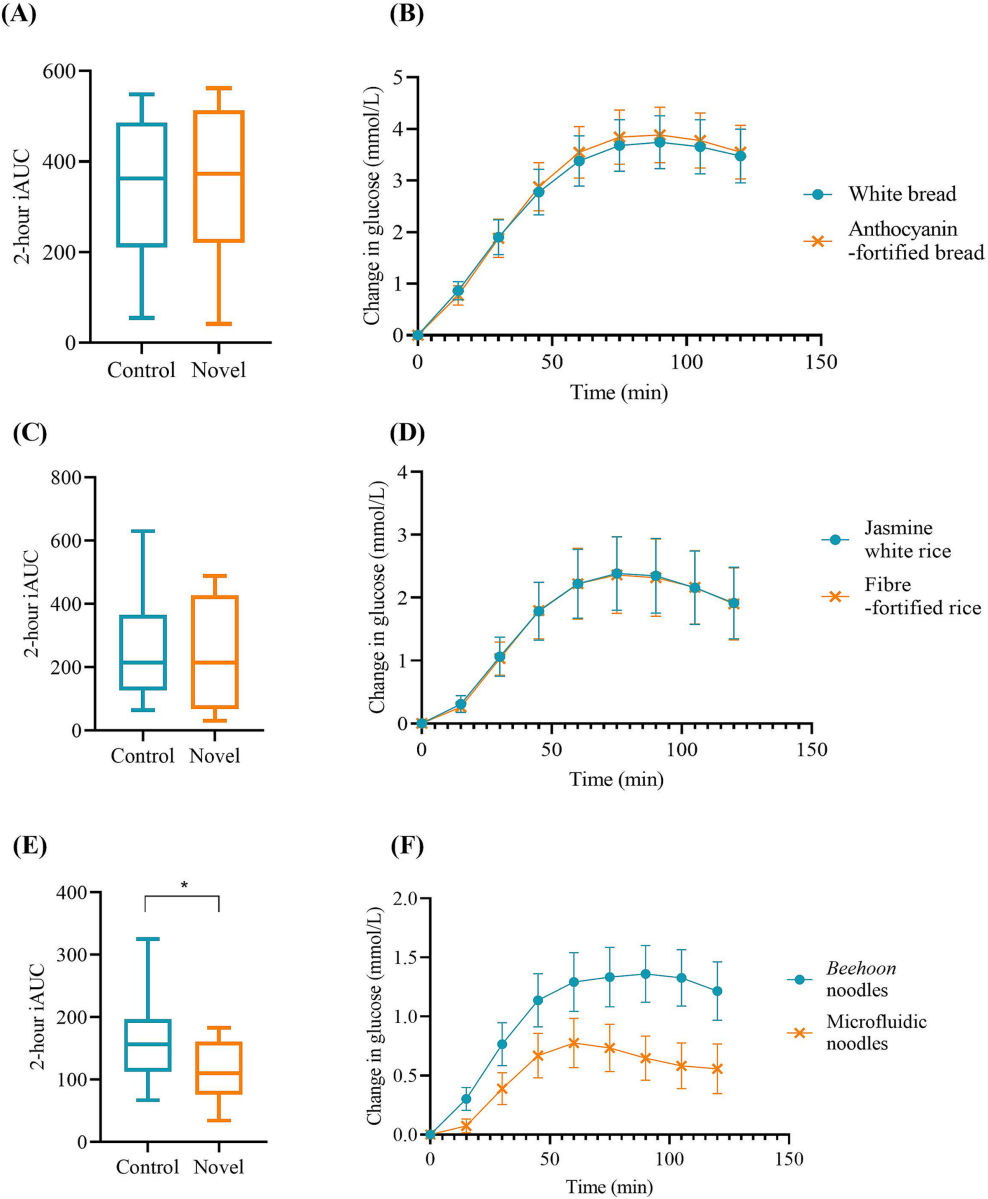
Postprandial glycemic responses to NVSFs in mixed meals. **(A)** 2-h iAUC and **(B)** change in glucose graph of white bread and anthocyanin-fortified bread; **(C)** 2-h iAUC and **(D)** change in glucose graph of jasmine white rice and fiber-fortified rice; and **(E)** 2-h iAUC and **(F)** change in glucose graph of beehoon noodles and microfluidic noodles. Values are presented as mean ± SEM. *p-value < 0.05 denotes significance.

The 2-h iAUC values, derived from CGM data, are presented in Figure 3. It summarizes the postprandial glycemic responses to each NVSF and its corresponding control. Figure 3A displays the iAUC values for the breakfast meal, showing that anthocyanin fortified bread (347.9 ± 44.5 mmol/L.min) and white bread (345.9 ± 40.0 mmol/L.min) elicited similar glycemic response. The difference was not statistically significant based on a paired t-test (*p* = 0.921, 95% CI = -41.22 to 45.31, Cohen’s *d* = 0.025). Corresponding glucose curves and peak times are shown in Figure 3B, where both breads reached their peak glucose concentration at 90 min. For the lunch meal, fiber-fortified rice (240.2 ± 41.9 mmol/L.min) and jasmine white rice (242.8 ± 40.4 mmol/L.min) also produced comparable glycemic responses, with no significant difference observed (*p* = 0.907, 95% CI = -50.71 to 45.35, Cohen’s *d = −*0.030; Figure 3C). Their respective glucose time curves are presented in Figure 3D, with both peaking at 75 min. Overall, no trend toward glycemic improvement was observed for the fortified bread or rice products, as evidenced by negligible effect sizes and non-significant differences. In contrast, the dinner meal (Figure 3E) showed a statistically significant (*p* = 0.046, 95% CI = −90.86 to −0.92, Cohen’s *d* = −0.544) reduction in 2-h iAUC with microfluidic noodles (114.3 ± 12.2 mmol/L.min) compared to *beehoon* noodles (160.2 ± 17.6 mmol/L.min). The effect size for this difference is considered medium, suggesting a meaningful practical difference in glycemic response between the two noodle types. Additionally, glucose peaked earlier and at a lower level with the microfluidic noodles—at 60 min with a change of 0.776 mmol/L—compared to *beehoon* noodles, which peaked at 90 min with a larger glucose change of 1.36 mmol/L (Figure 3F).

### 3.4 Joint display table

Table 6 presents a joint display of both qualitative and quantitative findings for each NVSF, integrating participants’ interview responses with CGM-derived glycemic data. This side-by-side format facilitates clearer interpretation of how participants’ subjective experiences align with their physiological glycemic responses, illustrating the value of the mixed methods approach.

**Table 6:** Joint display table of mixed methods results.

Novel staple food	Control staple food	Glucose attenuation effect in mixed meal	Modification method	Sensory properties		Price and willingness to pay	Healthfulness
				Appearance	Taste and Texture		
Anthocyanin-fortified bread	White bread	*Not significant* (*p* > 0.05)	Fortification	Visibly different in color Color associated with health properties Unique/novel Unappealing Similarities with local foods such as *pulut hitam*	No notable difference in taste as compared to control	Anticipation of a higher price stems from the recognition of special production costs and the use of premium ingredients Willingness to pay a higher price varies among individuals Among those willing to pay more: An acceptable increment of 10–20% or double the cost Readiness to pay more for health benefits Among those unwilling to pay more: Belief that prices should be comparable to conventional staples Consideration for lower prices, especially for seniors and individuals with low incomes who have diabetes Availability of cheaper alternatives in the market Preference for consuming smaller quantities of better-tasting and more affordable conventional staples	Healthfulness serves as motivational factor for consumption of NVSFs Improvement in blood glucose levels from personal measurements Should be backed by scientific evidence Belief NVSFs have a role to play in diabetes management by providing healthier staple food alternatives Despite high regard of health benefits, only willing to partially adopt NVSFs into diet
Fiber-fortified rice	Jasmine white rice	*Not significant* (*p* > 0.05)	Fortification	No notable difference in appearance from white rice Perceived as ‘bad’ for glucose regulation	No notable difference in taste as compared to control		
Microfluidic noodles	*Beehoon* noodles	*Significant* (*p* < 0.05*)	Carbohydrate reduction	Odd-looking Resembles Asian noodles such as *mee sua* and *beehoon*	Tasteless Rubbery Plastic Difficult to chew		

## 4 Discussion

### 4.1 Key findings

The expectations and perceptions of individuals with T2D toward NVSFs and their feasibility as alternative staple foods were explored and examined in this study. The qualitative analysis of interviews revealed that participants’ acceptance of the NVSFs was primarily influenced by their prior dietary choices as individuals with T2D, prior perceptions, expectations, and their visual first impressions of the NVSFs. The trade-off between sensory and health properties, price and willingness to pay, NVSF modification methods, as well as other socio-economic factors for regular consumption also influence acceptance. However, postprandial glycemic responses from 2-h iAUC of the test meals revealed only the meal with microfluidic noodles exhibited a significant improvement in 2-h iAUC relative to its control meal.

### 4.2 Preferences and familiarity

Fundamentally, the staple foods preferred to be modified by the participants represented the staple foods commonly consumed in their households: bread, rice, and noodles. This was expected, as modifications of these staples may improve their carbohydrate quality and allow for their continued consumption while promoting the participants’ well-being as individuals with T2D (4, 28). Additionally, rice was the top choice of staple food for majority of the participants. This also provided insight into the type of staple foods that should be targeted for modifications for health purposes.

When first introduced to the NVSFs, participants often related them to everyday foods they regularly consumed. Familiarity of foods may impact an individual’s attitude toward the foods, shaping values, beliefs, and norms relating to the food item. Therefore, NVSFs maintaining familiarity with minimal changes in sensory properties tended to be more attractive (29–31), and any sensory changes from conventional staples in NVSFs may evoke skepticism among the participants. Interestingly, a small subset of participants embraced the novelty of the anthocyanin-fortified bread, viewing its dark color as a positive attribute that set it apart from typical staple foods, signifying healthfulness and uniqueness. In contrast, the ‘normal’ appearance of fiber-fortified rice was also linked to the ‘bad’ reputation of white rice which was considered by many as one of the main causes of blood glucose surges. This finding was unexpected since the novel appearance of the anthocyanin-fortified bread was expected to potentially evoke feelings of uncertainty and doubt, while the conventional appearance of the fiber-fortified rice was expected to elicit feelings of security and comfort. These responses also reflected participants’ perceptions of existing conventional staples and how they believed these staples would impact their blood glucose levels.

### 4.3 Sensory vs. health trade-off

Notably, in post-intervention interviews, a trade-off between sensory and health properties was observed, a common conflict in numerous studies (32–35). Prior to the study, participants reported difficulties in restricting carbohydrate intake, suggesting that this may not be a sustainable diet approach for individuals with T2D. The introduction of NVSFs, on the other hand, was presented as diabetic-friendly alternatives of staple foods, potentially resolving the challenges associated with carbohydrate restriction. Furthermore, health claims made on conventional carbohydrate-rich staple foods which may have a ‘reduced health image’ may be inherently portrayed as ‘healthier’ choices, suggesting that the type of product featuring the health claim may also play a role in influencing an individual’s trust toward the claim or perceived health image (36). Thus, foods like NVSFs with health images or claims that address experienced diseased states may appear more attractive (37).

However, their consideration for NVSF regular consumption was still hindered by its taste and texture, in particular that of the microfluidic noodles. This further reiterates taste and texture as crucial sensory properties in the acceptance of new or novel foods and for their continued usage (16, 38–40). Despite some of their initial expectations to compromise on taste, participants remained highly critical of sensory properties (for microfluidic noodles), illustrating the extent to which such compromises in taste would be tolerated despite their perceived health properties, as similarly reported by others (41, 42). The implications of this trade-off for product formulation and consumer integration warrant further discussion and are explored in greater detail in Section 4.5.1.

### 4.4 Glycemic outcomes and product efficacy

Continuous glucose monitoring revealed only the meal with microfluidic noodles, but not anthocyanin-fortified bread or fiber-fortified rice, had exhibited a significant improvement in postprandial glycemic responses. We had initially hypothesized that the combination of macronutrients and functional ingredient fortification (i.e., anthocyanin- and fiber-fortification) could be an efficacious dietary strategy to affect postprandial glucose outcomes. This was based on existing studies that had demonstrated the impact of individual dietary constituents on postprandial glycemic responses. Proteins had exhibited an insulinotropic effect which accelerated the rate of glucose clearance in the blood (43). Dietary fibers and fats were noted to delay gastric-emptying rate and release the gastric inhibitory peptide (GIP) which promoted insulin secretion (44–46). Existing postprandial studies have also demonstrated improved glycemic responses through the intake of anthocyanin- and fiber-rich carbohydrate challenges *in vivo* (47–49). Unlike these studies that have only focused on individual food items, we evaluated the test foods as a mixed meal comprising standardized side dishes of protein, dietary fiber and fat—a more realistic setting akin to daily diets. The lack of efficacy of anthocyanin- and fiber-fortification in our study could be attributed to the confounding effect of macronutrients on postprandial glucose homeostasis when consumed as a meal. While Meng et al. (50) had initially hypothesized that additional macronutrients would impact postprandial glycemic responses, the investigators had concluded largely no significant changes to postprandial glucose outcomes when carbohydrate-rich foods are consumed as mixed meals of varying protein, fat, and fiber contents. Beyond meal compositions, Song et al. (51) highlights that individual responses to carbohydrates and mixed meals could vary greatly based on physiology, reporting coefficient of variances up to 68% for postprandial glycemic responses to different carbohydrate-rich mixed meals. These findings collectively indicate not only the confounding effects by macronutrients and individual variability which remain partially unaddressed in literature but also suggest potential challenges in postprandial glycemic predictions of actual eating occasions.

Despite this, a significant decrease in postprandial glycemic responses was still observed for microfluidic noodles. This can be attributed to its modification method involving the reduction of available carbohydrates, which would result in a lower amount of carbohydrates being metabolized in the test meal, in contrast to the anthocyanin-fortified bread and fiber-fortified rice meals which contained similar amounts of available carbohydrates as their respective controls. The amount of available carbohydrates has also been reported to be associated with T2D (52) and decreasing carbohydrate intake in a low-carbohydrate diet has been shown to improve glycemic control among individuals with T2D (53–55).

### 4.5 Challenges and recommendations for NVSF integration

#### 4.5.1 Palatability

Despite being preferred for their taste and texture, the anthocyanin-fortified bread and fiber-fortified rice did not meet participants’ high expectations for postprandial glycemic improvements. In contrast, the microfluidic noodles, which showed more favorable glycemic responses, were largely reported as unpalatable. This juxtaposition underscores a practical dilemma in NVSF development—achieving metabolic benefits while maintaining sensory appeal. The palatability of a staple food is crucial for promoting long-term, regular incorporation into the diet as highly palatable foods act as strong incentives for consumption and are often preferred over others (56). Therefore, the long-term incorporation of microfluidic noodles into the diet still poses as a challenge, despite the significant improvements in postprandial glycemic responses. Relying on an individual’s willingness to compromise product palatability for health purposes has also been reported as a speculative and risky approach in moderating food intake and eating behaviors (35). Hence, recognizing taste as a fundamental component of sensory cues is essential when promoting the consumption of healthier foods (56).

The dichotomy between health and consumer acceptance in the context of these NVSFs could be attributed to the methods used in the development process. Conventional carbohydrate-rich staple foods are recognized for their characteristic starchy taste. Modifications through fortification typically involve adding additional ingredients to the staple food while retaining its starchiness. This may explain the minimal differences in perceived taste and texture between the anthocyanin-fortified bread and the fiber-fortified rice and their respective controls. Nevertheless, modification of staple foods through fortification should not be entirely disregarded as they still serve as potential tools in improving blood glucose levels, as seen in other studies (47, 48). Fortification may also enhance the nutritional profile of the staple food which may offer other benefits such as the supplementation of micronutrients to achieve optimal blood biomarker levels and the reduction of deficiencies (57, 58). On the other hand, modification through a drastic reduction of available carbohydrates would greatly decrease the carbohydrate content of the staple food, potentially eliminating the perception of starchiness that may have contributed to the poor acceptance of taste and texture for the microfluidic noodles. Our findings suggest that near-complete carbohydrate replacement within a carbohydrate-based food matrix may be unsuitable, and that partial substitution offers a more practical and consumer-acceptable alternative. Multiple studies involving the development of modified carbohydrate staple foods adopted a partial substitution of flour with fiber or proteins, instead of a complete replacement, to ensure that product quality and consumer acceptance were met (59–61). This was attributed to the lack of significant changes in physicochemical and sensorial characteristics resulting from the partial substitution of carbohydrates in the modified food formulations. Further investigation into different formulations of macronutrients to be applied in carbohydrate reduction strategies would be necessary to validate a consumer-centric and functional carbohydrate product design. Moving forward, future product development and public health messaging should prioritize formulations that not only deliver metabolic benefits but also meet consumer taste expectations. Designing carbohydrate-reduced staple foods with a consumer-centric approach is essential to ensure both effectiveness and real-world dietary integration.

#### 4.5.2 Inclusivity and everyday use

More measures can be implemented to improve the adoption rate of NVSFs, not only among individuals with T2D but also among their families. Consistent with studies showing the importance of familial influences (62, 63), family support is key for continued use of new or novel foods. Importantly, the portrayal of these foods as being reserved for individuals with T2D acts as a deterrent for their adoption. Our results suggest that communication efforts should be careful when portraying NVSFs as being specifically tailored for individuals with T2D. NVSFs can be promoted to effectively illustrate that they can be readily enjoyed by everyone, both individuals with and without diabetes, as part of local delicacies. Recommendations on ways to prepare and consume the food can also be provided, as suggested by participants. NVSFs can also be offered in a wider range of stores or convenience shops situated in neighborhoods for easy access.

#### 4.5.3 Balancing perceived value and affordability

The perceived health benefits also led many participants to anticipate higher prices of NVSFs, a view that participants continued to hold in post-intervention interviews. This was also an anticipated outcome, as reported by similar studies (64–66). However, the willingness to pay these higher prices was subjective and likely shaped by intrinsic factors such as income, education level, and age (67, 68). While reasons for the willingness to pay more was primarily driven by perceived health benefits, unwillingness to pay more was mainly driven by financial considerations. It should also be noted that the novel fiber composition blend (5ibrePlus™, Alchemy Foodtech Pte. Ltd., Singapore) used in the production of the fiber-fortified rice is available for purchase in Singapore supermarkets. The price of 1 cup (150 g) of uncooked white rice (5 kg Thailand White Rice, Fairprice Group, Singapore) is SGD $0.23, while the price of 1 cup (150 g) of uncooked brown rice (5 kg Thailand Brown Unpolished Rice, Fairprice Group, Singapore) is SGD $0.40. However, the total cost of fortification of the aforementioned white rice with 15 g fiber composition blend (the amount of fiber blend recommended for 1 cup of uncooked rice) would be approximately SGD $0.87. Unfortunately, this does not meet the 10–20% or double price increment that participants were willing to pay for an NVSF, in comparison to the brown rice. To address this gap, early-stage pricing strategies could include short-term price promotions or discount schemes that preserve perceived product value while reducing cost barriers. Such approaches may be more effective than permanent price reductions, as they maintain the value of the product (69). These strategies could also serve to ease NVSF products into the competitive food market, allowing the consumers to trial them without immediately rejecting them based on cost (69). In the longer term, broader public health measures—such as taxing unhealthy foods and offering subsidies or tax incentives for healthier alternatives like NVSFs—may improve encourage consumers to make healthier choices (70). Therefore, future pricing strategies should consider both perceived value and affordability to enhance the long-term feasibility and accessibility of NVSFs among the general public.

### 4.6 Market landscape

In Singapore, several diabetes-friendly products, such as AuroraFood’s low-GI baked goods and Glucerna nutritional products, are marketed to support glycemic control. Moreover, the fiber-fortified rice used in this study is already commercially available, while the anthocyanin-fortified bread has comparable alternatives in the market. However, broader consumer adoption of all these diabetes-friendly products remains limited, potentially due to factors such as cost, unfamiliarity, and suboptimal sensory appeal, as highlighted in our findings. The microfluidic noodles, while still under development, represent an innovative approach to glycemic management but require significant improvements in palatability to enhance consumer acceptance. While the growing availability of diabetes-friendly products reflects market interest, our findings suggest that availability alone is insufficient. Therefore, successful integration of these diabetes-friendly foods requires strategies that address key barriers, as discussed in Section 4.5.

### 4.7 Strengths and limitations

The strength of this study is derived from the utilization of a mixed methods approach, which acknowledges both the subjectivity of qualitative analysis and the objectivity of quantitative analysis, thereby facilitating the mutual reinforcement of the two datasets. This amplifies the interpretations derived, contributing to a comprehensive understanding of the acceptance and feasibility of NVSFs among individuals with T2D. Furthermore, the crossover design of this study also minimizes potential confounding and variability factors that may arise among participants. Participant compliance to test meals was also improved through the provision of the meals free of cost and the submission of a photograph of their empty food containers following meal consumption.

However, several limitations must be acknowledged to contextualize our findings. First, this study had a relatively small sample size (*n* = 16). While appropriate for an exploratory study, this limits the generalizability of the results to the broader population of individuals with T2D. Nonetheless, the outcomes provide valuable preliminary insights and lay the foundation for future larger-scale trials. The study duration was also relatively short (5 weeks), and no follow-up was conducted to explore whether participants would continue consuming NVSFs beyond the study period. As such, future research should explore the long-term sustainability, adherence, and metabolic impacts of incorporating NVSFs into habitual diets.

Second, certain aspects of the intervention design may have shaped participant experiences. The use of standardized side dishes across both intervention periods, which was implemented to control for macronutrient consistency, was noted by some participants as reducing meal variety and flavor. This lack of variety may have undermined the participants’ overall experiences and perceptions toward the NVSFs. Similarly, the absence of restrictions during the snacking window—aimed to mirror real-world behaviors—may introduce potential second-meal effects (71). Participants were also not blinded to the intervention, which may have influenced subjective feedback regarding their perceptions and experiences of the NVSFs and their dietary habits during the novel intervention. However, postprandial glycemic responses were objectively measured using continuous glucose monitoring, minimizing reporting bias. Novelty bias may have also played a role in shaping participants’ reactions—both positive and negative—to the intervention foods, as their unfamiliarity or uniqueness may have temporarily influenced perceptions of palatability or acceptability. This effect may not fully represent sustained preferences over time.

Third, the demographic profile of the participants may limit broader applicability of the findings. The average age of the recruited participants was 57.9 ± 9.45, with younger adults underrepresented. This is noteworthy, as age differences may influence food preferences and openness to NVSFs. For example, generational food habits may shape familiarity and comfort with traditional staples, with older adults potentially showing greater resistance to change (72, 73) compared to younger individuals. Nonetheless, evidence also suggests that older adults often prioritize health benefits and may therefore be more receptive to functional foods that address specific health concerns (74). Additionally, all participants were of Chinese ethnicity, which helped minimize potential ethnicity-related confounding effects associated with postprandial glycemic responses. However, this limits the applicability of the findings to other cultural groups, particularly within Singapore’s multi-ethnic context. Individuals from other ethnic backgrounds may exhibit different glycemic responses based on genetic variation (75), and may hold differing perceptions of the NVSFs shaped by cultural food practices. For instance, staple food preferences, food preparation methods, and broader dietary norms can vary significantly among ethnic groups in Singapore (76), all of which can affect both attitudes and preferences toward NVSFs. Future studies involving more diverse populations would provide richer insight into how age and cultural-related differences could affect NVSF acceptance and feasibility.

Finally, other demographic factors such as education level, socioeconomic status and marital status were not collected. While not central to the present analysis, these variables may provide valuable context regarding dietary behavior and openness to novel foods and could be included in future studies to inform more tailored interventions.

## 5 Conclusion

In this study, we examined the expectations and perceptions of three distinct NVSFs (anthocyanin-fortified bread, fiber-fortified rice, and microfluidic gel noodles) and their feasibility as alternative staple foods among individuals with T2D. Our findings revealed that participants’ prior dietary choices as individuals with T2D, prior perceptions, expectations, and their visual first impressions of NVSFs, the trade-off between sensory and health properties, price and willingness to pay, NVSF modification methods, and other socio-economic factors were the primary themes influencing participants’ acceptance. However, analysis from postprandial glycemic data revealed only the meal with microfluidic gel noodles exhibiting a significant improvement in 2-h iAUC relative to its control, despite it receiving substantial criticisms for its sensory properties. Using a mixed methods approach, our results suggest a conflict between health and taste concerning the NVSFs in this study. This was likely attributed to their modification methods, which involved the drastic reduction of available carbohydrates for microfluidic noodles, leading to the absence of the typical ‘starchy’ taste associated with carbohydrate-rich staple foods. As such, the innovation of an NVSF with favorable and familiar taste and texture properties modified through the reduction of available carbohydrates approach may boost its acceptance and feasibility as a good alternative form of staple food among individuals with T2D.

## Data Availability

The original contributions presented in the study are included in the article/Supplementary material, further inquiries can be directed to the corresponding author.
