# Supplementary material for: Acceptance and feasibility of novel staple foods among individuals with type 2 diabetes in Singapore: a mixed methods study
Source: Front Nutr. 2025 Jun 11;12:1594890. doi: 10.3389/fnut.2025.1594890 (PMC12189021; doi:10.3389/fnut.2025.1594890)
Supplement: Supplementary file 1 [file Table_1.DOCX]

Supplementary Material

# Supplementary Material 1

# Preparation of anthocyanin-fortified bread and control white bread.

The ingredients used comprised 1.2 g of salt, 4 g of sugar, 3 g of shortening, 1 g of instant dry active yeast, and 59 g of water for every 100 g of bread flour. To prepare the anthocyanin-fortified bread, 4 g of black rice anthocyanin extract (Hubei Zixin Biological Technology Co Ltd., Hubei, China) was integrated into the ingredients. All the components, excluding water, were mixed in an electric mixer (KitchenAid, Michigan, USA) for 1 minute. After thorough mixing of the ingredients, water was added, and the mixture was further combined for an additional 1 minute at low speed, followed by an additional 5 minutes at medium speed. The resulting dough was allowed to rest for 15 minutes before being divided and shaped into 75 g boules. These boules were then proofed at 40°C and 85% relative humidity for 70 minutes and baked in the oven at 200°C for 8 minutes.

# Supplementary Material 2

# Preparation of microfluidic noodles.

Briefly, 2% w/v food-grade sodium alginate solutions (Phoon Huat Pte. Ltd., Singapore) and 12% w/v soy protein isolate solutions (Fujipro, Fuji Oil Holdings Inc., Osaka, Japan) were prepared by blending the powders with water using a handheld blender (MR 400, Braun GmbH, Kronberg, Germany). Each solution was loaded into 20 mL plastic syringes. Mechanical syringe pumps (NE-4000, New Era Pump Systems, Inc., Farmingdale, NY, USA) were used to extrude the soy protein isolate solution as the inner fluid and the sodium alginate solution as the outer fluid through a microfluidic co-flow device as per methodology outlined by Lin et al. (2022). This process resulted in the production of noodle-like microfluidic assemblies, which were directly extruded into a 3% w/v calcium chloride (Phoon Huat Pte. Ltd., Singapore) bath to facilitate the gelation of the extruded microfluidic gel noodles.

# Supplementary Material 3

**Table S1.** Sample of guided interview questions.

| Pre-Intervention |
| --- |
| 1. What is the most consumed staple food in your household? 2. What do you understand by the term ‘novel staple food’? 3. How have your experiences been with novel staple foods? 4. Which staple food would you like to be modified? Why? 5. What would attract you to novel staple foods? 6. [Interviewer to present NVSF samples] What are your thoughts about the (novel) bread, rice, and noodle? |
| Post-Intervention (Novel) |
| 1. What were your experiences with using the staple foods in the intervention? 2. Were there any problems or unanticipated issues you faced while consuming these foods? 3. Were there any factors that helped/motivated you to consume these foods? 4. In your opinion, what could be done to make your experiences better? 5. What are your thoughts about consuming these foods on a regular basis if they were available in the market? 6. Which novel staple food are you most likely to accept? 7. What are some concerns you have about replacing your everyday staples with these novel staples? 8. What are your thoughts about how much these foods should cost? 9. Could you share your personal experiences with diabetes? 10. What are your thoughts about the role novel staple foods can play to help better manage diabetes? |
| Post-Intervention (Control) |
| 1. What were your experiences with using the staple foods in the intervention? 2. Were there any problems or unanticipated issues you faced while consuming these foods? 3. Were there any factors that helped/motivated you to consume these foods? 4. In your opinion, what could be done to make your experiences better? 5. What are your thoughts about consuming these foods on a regular basis if they were available in the market? |

# Supplementary Material 4

**Table S2.** Change in HbA1c and weight across interventions.

|  | **HbA1c (%)** | | | | **Weight (kg)** | | | |
| --- | --- | --- | --- | --- | --- | --- | --- | --- |
|  | Control | | Novel | | Control | | Novel | |
| Subject | Before | After | Before | After | Before | After | Before | After |
| 1 | 6.8 | 6.9 | 6.9 | 6.7 | 65.0 | 65.8 | 65.6 | 65.9 |
| 2 | 6.5 | 6.4 | 6.5 | 6.3 | 76.7 | 77.1 | 76.3 | 78.5 |
| 3 | 7.3 | 7.5 | 7.6 | 7.7 | 50.7 | 50.9 | 52.6 | 51.1 |
| 4 | 7.8 | 8.0 | 8.1 | 8.0 | 89.7 | 88.1 | 89.3 | 88.7 |
| 5 | 7.5 | 7.4 | 7.4 | 7.4 | 75.8 | 75.0 | 76.6 | 77.0 |
| 6 | 6.2 | 6.5 | 6.4 | 6.4 | 40.7 | 40.7 | 41.1 | 40.7 |
| 7 | 7.8 | 7.4 | 7.3 | 7.0 | 58.4 | 58.0 | 58.3 | 57.4 |
| 8 | 6.7 | 7.0 | 6.8 | 6.8 | 71.9 | 71.5 | 71.6 | 71.2 |
| 9 | 7.0 | 7.1 | 7.0 | 7.0 | 50.8 | 50.7 | 51.0 | 50.7 |
| 10 | 6.8 | 6.9 | 6.7 | 6.9 | 68.4 | 68.6 | 68.9 | 68.9 |
| 11 | 8.0 | 8.1 | 8.1 | 7.9 | 71.2 | 71.8 | 71.1 | 71.6 |
| 12 | 6.3 | 6.2 | 6.4 | 6.2 | 55.7 | 55.4 | 56.4 | 56.1 |
| 13 | 7.8 | 7.7 | 8.0 | 7.5 | 62.6 | 60.8 | 62.0 | 61.2 |
| 14 | 7.4 | 7.6 | 7.8 | 8.3 | 127.3 | 128.8 | 128.4 | 128.4 |
| 15 | 8.2 | 7.8 | 7.7 | 7.6 | 89.3 | 87.5 | 87.8 | 87.9 |
| 16 | 7.1 | 6.8 | 6.9 | 6.7 | 107.1 | 106.0 | 106.4 | 105.9 |
| Mean | 7.15 | 7.17 | 7.18 | 7.11 | 71.9 | 71.6 | 72.0 | 71.8 |
| SD | 0.62 | 0.56 | 0.61 | 0.63 | 21.7 | 21.7 | 21.5 | 21.7 |
